# Synthesis of Large-Scale Single-Crystalline Monolayer WS_2_ Using a Semi-Sealed Method

**DOI:** 10.3390/nano8020100

**Published:** 2018-02-11

**Authors:** Feifei Lan, Ruixia Yang, Yongkuan Xu, Shengya Qian, Song Zhang, Hongjuan Cheng, Ying Zhang

**Affiliations:** 1 Institute of electronic information and engineering, Hebei University of Technology, Tianjin 300401, China; lanfeifei0601@126.com (F.L.); qianshengya@163.com (S.Q.); 2 China Electronics Technology Group Corp 46th Research Institute, Tianjin 300220, China; orientation@126.com (Y.X.); zhangsong@126.com (S.Z.); chenghongjuan@163.com (H.C.); zhangying@126.com (Y.Z.)

**Keywords:** semi-sealed, CVD, WS_2_, Raman, AFM

## Abstract

As a two-dimensional semiconductor, WS_2_ has attracted great attention due to its rich physical properties and potential applications. However, it is still difficult to synthesize monolayer single-crystalline WS_2_ at larger scale. Here, we report the growth of large-scale triangular single-crystalline WS_2_ with a semi-sealed installation by chemical vapor deposition (CVD). Through this method, triangular single-crystalline WS_2_ with an average length of more than 300 µm was obtained. The largest one was about 405 μm in length. WS_2_ triangles with different sizes and thicknesses were analyzed by optical microscope and atomic force microscope (AFM). Their optical properties were evaluated by Raman and photoluminescence (PL) spectra. This report paves the way to fabricating large-scale single-crystalline monolayer WS_2_, which is useful for the growth of high-quality WS_2_ and its potential applications in the future.

## 1. Introduction

Two-dimensional material such as transition mental dichalcogenides (TMDCs) and black phosphorus have attracted great interest for their unique physical properties [1,2,3,4,5,6,7,8,9,10,11], especially for TMDCs. In contrast to zero-bandgap graphene [12], TMDCs are two-dimensional semiconductor with available bandgap when in bulk [1,8]. With the reduction of the thickness, the bandgap transforms from an indirect to a direct one. Meanwhile, the bandgap can be adjusted through the sythesis of the alloy with different stoichiometry based on TMDCs. This intrinsic adjustable bandgap and flexibility allows them to be used in optoelectronic and nanoelectronic devices. There have been plentiful and inventive works focused on the synthesis methods [13,14,15], and their optical [16,17], electronic [18,19,20], and catalysis properties [21,22].

Among the TMDCs, MoS_2_ and WS_2_ are two perfect examples. Now, a great deal of research is focused on the study of MoS_2_. In fact, WS_2_ is a more promising transition mental dichalcogenides for electronics [23], because of its superior mobility and its chemical robustness [24]. However, compared to MoS_2_, the research of WS_2_ is a long way away from being enough, especially with regard to the synthesis of large-scale single-crystalline WS_2_ with monolayer. Mechanical exfoliation has been extensively used to obtain an atomically thin WS_2_ film for the research of its related properties, but the size of the film obtained by this method is too small to study the devices based on two-dimensional WS_2_. Most recently, CVD has been successfully used for the synthesis of MoS_2_ film at large scale [25,26,27,28,29]. For this reason, CVD has also been considered an efficient method for the growth of WS_2_. The growth process consists of the sulfidation of WO_3_ powders through S vapor. Although monolayer WS_2_ has been synthesized with a size of hundreds of microns by CVD [30,31,32], the uniformity and the repeatability is really poor. The main reasons for these results are the high melting point of WO_3_ powders, and the fact that the growth process is very sensitive to the sulfidation rate. In this paper, we report the synthesis of single-crystalline triangular WS_2_ film with large size through a semi-sealed CVD method. A semi-sealed quartz boat was used to enhance the partial pressure of WO_3_. With a higher partial pressure of WO_3_, the WS_2_ monolayer film with the grain size of more than 400 μm was obtained. This paves the way to the growth of monolayer WS_2_ and related TMDCs with large grain size.

## 2. Growth Process

Triangular WS_2_ monolayer films were grown by CVD in a horizontal furnace. High-purity Ar was the carrier gas with a flow rate of 100 sccm, 3 mg WO_3_ powders were placed into a small quartz boat as W source, high-purity S powders were S source, and Al_2_O_3_ was chosen as the substrate, face-down above the WO_3_ powders. Compared to the lower growth temperature of MoS_2_, the growth temperature of WS_2_ was as high as 1050 °C, with a pressure of 10 mbar.

## 3. Results and Discussion

**Figure 1:**
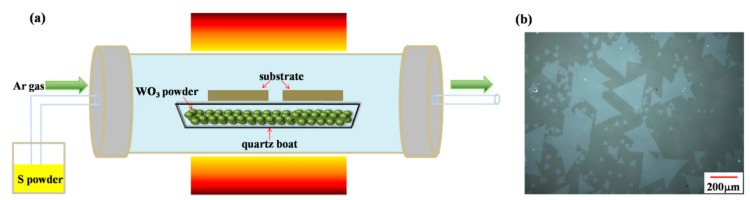
(**a**) Schematic diagram of CVD growth system; (**b**) optical image of WS_2_.

Similar to the growth of MoS_2_, the synthesis of WS_2_ is very sensitive to the sulfidation rate; too fast or too slow are both detrimental to the growth of large-scale WS_2_ film. An effective way to solve this problem is to control the evaporation rate of the S source. In order to control the temperature and evaporation rate of the S source, S powders were placed into an independent stainless-steel cylinder out of the furnace with a heating belt and a thermocouple to control the temperature. The integral structure is shown in Figure 1a. Through this system, we successfully obtained triangular monolayer WS_2_ film; the edge length of the triangles was about 150 µm, as shown in Figure 1b.

In the course of conducting this research, we found it difficult to obtain triangles with larger size. The reason for this phenomenon is the lower vapor pressure of WO_3_. As we know, the melting point of WO_3_ is as high as 1300 °C; such a high melting point makes it difficult to enhance the partial pressure of WO_3_ vapor. A lower pressure of WO_3_ vapor will result in a shortage of the W source on the surface of the substrate. So we have to enhance the partial pressure of WO_3_ vapor to enlarge the size of WS_2_ film. The most efficient way to enhance the partial pressure of WO_3_ is to reduce the pressure of the furnace during the growth of WS_2_. A low pressure can lower the melting point of WO_3_ to increase the partial pressure of WO_3_. However, in this condition, the transport speed of the S vapor will also increase. This will increase the sulfidation rate. As we know, a high sulfidation rate is adverse for the migration and diffusion of the atoms and molecules on the surface of the substrate. This will make it difficult for the acquisition of single-crystal WS_2_ film with large size. So we need to find an efficient way to increase the partial pressure of WO_3_ and keep the transport of S vapor under a low speed.

**Figure 2:**
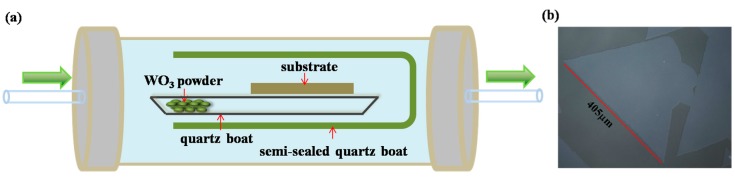
(**a**) Semi-sealed equipment schematic diagram; (**b**) optical microscope of 405μm monolayer WS_2_ film.

In this paper, a semi-sealed quartz boat was used to enhance the partial pressure of WO_3_ vapor. The small quartz with WO_3_ powders and the substrate were put into a semi-sealed quartz boat, and the substrate was placed downstream of the W source to reduce the nucleation centers at the beginning of the growth to enlarge the size of the single-crystalline triangles. The distance between the W source and the substrate was 3–5 cm, as shown in Figure 2a. During growth, WO_3_ vapor was limited in such a semi-sealed quartz boat. The partial pressure of the WO_3_ vapor can be greatly enhanced relative to the pressure of the whole furnace. Meanwhile the pressure of the furnace can be kept at a higher pressure to reduce the transport speed of the S vapor. With this method, the length of the largest triangular WS_2_ increased to about 405 μm, as shown in Figure 2b.

**Figure 3:**
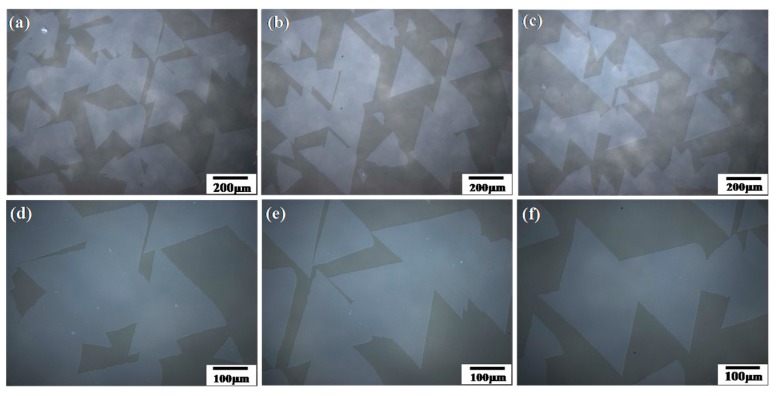
Optical images of the triangles at different locations with different magnifications: (**a**–**c**) are the images with a magnification of 10×; (**d**–**f**) are the images with a magnification of 20×.

Figure 3 shows the optical microscopy images of triangular WS_2_ films. Most of the films are monolayer, the size of the triangles enlarged to more than 300 μm on each side, and the nucleation density reduced obviously. Furthermore, from the optical images, we can see that the orientation of the triangles was not complete disorder. Many of the triangles present a slightly epitaxial growth mechanism. This maybe results from the high growth temperature of 1050 °C and an annealing process of the sapphire before sulfidation. According to the research of the Kis group [33], annealing of the sapphire is helpful for the growth of WS_2_ triangles with the same orientation. This results from the enhanced Van der Waals force. Additionally, the annealing of the sapphire is helpful for the reduction of nucleation density because of the clean surface of the substrate. These results provide a new method for the growth of continuous single-crystal WS_2_ monolayers.

**Figure 4:**
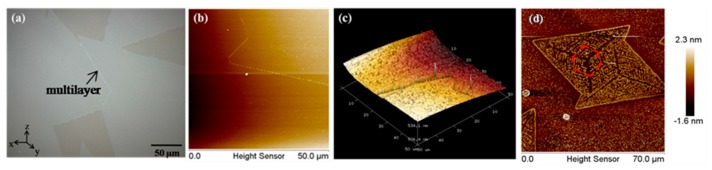
(**a**) optical image on the grain boundary; (**b**) 2D image of the grain boundary by atomic force microscope (AFM); (**c**) 3D image of the grain boundary by AFM; (**d**) outline of the film by AFM.

During the research, we found an interesting thing, as shown in Figure 4a. On the grain boundary of two connecting triangles with great difference in torsion angles, the film is multilayer. However, the multilayer film is only concentrated on the grain boundary. This can also be seen through AFM, Figure 4b,c shows the 2D and 3D image of the film. Through the image, we can see that the film is multilayer on the grain boundary. Through the research, we find that this phenomenon can be observed only on the two connecting triangles with great difference in torsion angles. For those triangles that do not connect with each other, or that connect with each other but with a small difference in torsion angles, this growth does not appear. This maybe results from the great difference of torsion angles between two connecting triangles. With a great difference in torsion angles, a grain boundary will appear on the interface of two different triangles. The appearance of the grain boundary will result in the disorder of the growth. This disorder growth will make a mismatching stitching between two different triangles, resulting in an overlapping growth of two triangular grains with different orientations. However, we need some more tests to prove it.

Additionally, we found an interesting thing during the AFM testing. The film appears to have obviously fallen off due to the scraping by the probe during the AFM testing. After the falling off of the film, we can clearly see the outline of the film, as shown in Figure 4d. From the image, we can see that the growth of the film begins at the centre of the triangles. The growth may be a symmetrical growth along the centre and the diagonal of the triangles.

**Figure 5:**
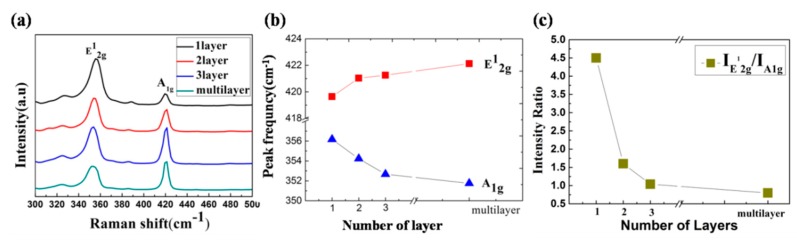
(**a**) Raman spectra with different WS_2_ thicknesses; (**b**) frequency change of A_1g_ and $E_{2g}^{1}$ with different thicknesses; (**c**) $I_{E_{2g}^{1}}/I_{A_{1g}}$ ratio with different layers.

Optical properties were charactered by Raman and PL spectra. Figure 5a presents the Raman peak of the triangles with different thicknesses. With the increasing of the number of layers, the Van der Waals force suppresses atom vibration, resulting in higher force constants, so the blueshift of A_1g_ corresponds to the predicted stiffening [34,35]. However, the $E_{2g}^{1}$ peak exhibits redshifts when increasing the number of WS_2_ layers. This suggests that long-range Coulombic interlayer interaction or the changing of the structure based on the stacking of different layers plays a major role [4,35]. The peak frequency and the ratio of $I_{E_{2g}^{1}}/I_{A_{1g}}$ are summarized in Table 1. With the increase in the number of WS_2_ layers, the ratios of $I_{E_{2g}^{1}}/I_{A_{1g}}$ decreased from 4.5 to 0.8, an obvious change. This can be used as an effective way to identify the WS_2_ films with different thicknesses.

**Figure 6:**
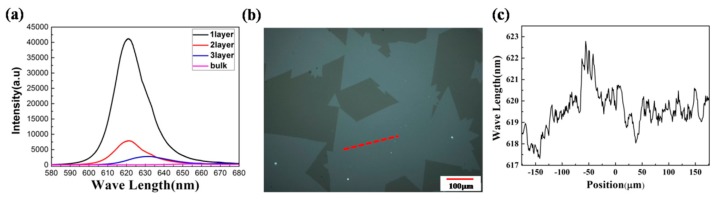
(**a**) Photoluminescence (PL) spectra of WS_2_ with different thickness; (**b**) optical image of WS_2_ and the red line is position for PL line scanning; (**c**) PL line scanning image.

Figure 6a shows the PL spectra at the same position as the Raman spectra. The PL spectra display an indirect to direct bandgap from multilayer to monolayer. With the decreasing of thickness, the intensity of PL peaks increases dramatically. The PL intensity is extremely weak in multilayer, consistent with an indirect bandgap semiconductor. Meanwhile, the increase of PL intensity implies the increase of direct interband transition with the decreasing of thickness. The peak moved to shorter wavelength with the decreasing of thickness, which indicates an increase in the bandgap, and reaches its maximum at the monolayer, which is about 2.0 eV. In order to investigate the differences of the photoluminecence properties between the edge and the other areas, as well as the grain boundaries, we choose a typical position on the surface of the film to perform PL line scanning. Figure 6b shows the position of the PL line scanning. According to Figure 6c, the PL peak did not changes obviously at different areas. This result indicates a high quality with a good uniformity of the film.

## 4. Conclusions

In conclusion, we grew monolayer single-crystalline WS_2_ triangles with large size using a semi-sealed CVD method. The largest triangle was about 405 μm in length. Many of the triangles present a slightly epitaxial growth mechanism. Raman spectra show that most of the triangles are monolayer. PL spectra indicate the good uniformity and high quality of the triangles. This method can be used for the growth of large-scale single-crystalline WS_2_ film.

## Figures and Tables

**Table 1:** Summary of the peak frequency for A_1g_ and $E_{2g}^{1}$ and the intensity ratio of the two peaks as a function of thickness with the excitation wavelength 514 nm.

λ_Exc_	Phonon Modes	1 Layer	2 Layer	3 Layer	Multilayer
514 nm	$E_{2g}^{1}$ (cm^−1^)	356.17	354.23	352.67	352.75
	A_1g_ (cm^−1^)	419.64	421.03	421.25	422.12
	$I_{E_{2g}^{1}}/I_{A_{1g}}$	4.5	1.6	1.04	0.8
